# Adverse Drug Reaction Monitoring in Multidrug-Resistant Tuberculosis Patients Receiving Bedaquiline and Delamanid-Based Regimen

**DOI:** 10.7759/cureus.30764

**Published:** 2022-10-27

**Authors:** Elisha Paikray, Priti Das, Manoranjan Pattnaik, Vedvyas Mishra

**Affiliations:** 1 Pharmacology, Utkal University, Cuttack, IND; 2 Pharmacology, Srirama Chandra Bhanja (SCB) Medical College and Hospital, Cuttack, IND; 3 Pulmonary and Critical Care, Srirama Chandra Bhanja (SCB) Medical College and Hospital, Cuttack, IND

**Keywords:** drug resistance, tuberculosis, delamanid, bedaquiline, adverse drug reaction

## Abstract

Background and objectives* *

Tuberculosis (TB) is an airborne contagious illness caused by *Mycobacterium tuberculosis*. Ineffective anti-TB medication prolongs and exasperates illness, promotes disease spread, increases the probability of developing resistance to treatment, and increases death rate. Bedaquiline (BDQ) and Delamanid (DLM) were conditionally made available in the treatment of multidrug-resistant TB (MDR TB). In drug-resistant TB patients, adverse drug reactions (ADR) management is essential to improve medication compliance. In addition, we performed this study since there are very few studies published on the analysis of ADR monitoring of BDQ and DLM-based regimen. This study was performed to study the spectrum of ADR in drug-resistant TB patients receiving BDQ and DLM-based regimen.

Methodology

The study was conducted over a period of 26 months, in a hospital's departments of pharmacology and pulmonary medicine. Pre-extensively drug-resistant (Pre-XDR) and XDR TB were established on the basis of cartridge-based nucleic acid amplification (CB-NAAT), line probe assay (LPA), drug susceptibility testing (DST), and bacteriological culture. Patients were prescribed with appropriate medicines at the initial visit and any adverse reactions to medication were assessed in the subsequent visit. The statistical analysis was done using frequency distribution procedure, chi-square test of independence. The significance level was set at p<0.05.

Results

It was revealed that there were as many as 24 types of ADRs manifested in different patients. The most frequent ADR was QTcF (corrected heart rate) prolongation.

Conclusion

The maximum number of patients had some form of ADR and the percentage was slightly higher in the BDQ group than in the DLM group.

## Introduction

Tuberculosis (TB) is an airborne contagious illness caused by *Mycobacterium tuberculosis* (MTB). Curable infectious infections like these are among the top 10 leading causes of mortality globally. In 2017, an estimated 10.4 million new cases of TB were reported globally, including 600,000 cases of rifampicin resistance and 490,000 cases of multidrug-resistant (MDR) TB (defined as TB resistant to rifampicin and isoniazid with or without resistance to other first-line treatments). The countries with the highest number of MDR TB cases (45% of the global total) are India, China, and the Russian Federation [1-4]. MDR TB is caused due to poor compliance with treatment. There are a variety of risk factors for acquiring an active tuberculosis case that include human immunodeficiency virus (HIV) infection, low socioeconomic status/poverty, alcoholism, homelessness, crowded living conditions, and diseases that impair the immune system. Ineffective anti-TB medication prolongs and exasperates illness, increases death, promotes disease spread, and increases the probability of developing resistance to treatment. As a result, it has a significant financial impact on both individuals and the health care system, with a greater number of pharmaceuticals with more serious side effects [3,4].

Bedaquiline (BDQ) and Delamanid (DLM), two new anti-TB medications discovered approximately five decades after rifampicin, were conditionally made available under the Control Access Program for use as a rear regimen in the treatment of multidrug-resistant TB.

BDQ is a diarylquinoline that acts by inhibiting mycobacterial adenosine triphosphate (ATP) synthase, interrupting energy production and breaking down the intracellular metabolism [5]. It is active against almost all MTB strains resistant to rifampin, isoniazid, streptomycin, ethambutol, and pyrazinamide. DLM is the first medicine of the nitro-dihydro-imidazooxazole family to be approved for the treatment of multidrug-resistant tuberculosis. It acts by inhibiting the synthesis of methoxy mycolic acid.

An effective approach is needed to track and report adverse drug events in order to develop safe medication practice guidelines for the treatment of drug-resistant tuberculosis (DR TB) [6]. In DR TB patients, adverse drug reaction (ADR) management is essential to improve medication compliance. Non-compliance with treatment has a significant financial impact on both patients and the healthcare system, resulting in the use of more drugs with more serious side effects. In addition, there are very few studies published on the analysis of ADR monitoring of BDQ and DLM-based regimen so this study was performed.

This study aimed to study the spectrum of ADR in drug-resistant TB patients receiving BDQ and DLM-based regimen. Also, the study aimed to assess the causality and severity of the monitored ADRs.

## Materials and methods

The study was conducted over a period of 26 months, in a tertiary care teaching hospital's departments of pharmacology and pulmonary medicine. Baseline data regarding patients' demographic profiles, medication regimen, and ADRs were collected by the principal investigator from the in-patient department of pulmonary medicine department and via telephonic conversation and were recorded in a pre-designed case record form. In the study, both male and female patients between the age of six and 18 years with DLM and above the age of 18 years with BDQ were included; patients who were HIV-infected, those who had type 2 diabetes, chronic obstructive pulmonary disease (COPD), and hypertension were included. Pregnant and lactating women, patients with torsades de pointes or cardiac ventricular arrhythmias, and those who were hypersensitive to the active ingredient or any of the excipients were excluded from the study. The Declaration of Helsinki's ethical criteria were adhered to at all times. Before beginning the study, the Institutional Ethics Committee of Srirama Chandra Bhanja Medical College and Hospital granted permission. All patients who agreed to participate in the trial provided written consent.

Pre-extensively drug-resistant (Pre-XDR) and XDR TB were established on the basis of cartridge-based nucleic acid amplification (CB-NAAT), line probe assay (LPA), drug susceptibility testing (DST), and bacteriological culture. All selected patients underwent clinical, serological, and laboratory tests before initiation of therapy. The case definition of the study of patients with pre-XDR TB was resistance to fluoroquinolones (Fq-r) or second-line injectables (SLI) resistance. XDR TB patients were resistant to both Fq and SLI [7,8].

Patients were prescribed appropriate medicines at the initial visit and any adverse reactions to medication were assessed in the subsequent visit. A total of 86 patients were included, among which 81 were pulmonary and five extrapulmonary TB patients. Sixty-five patients received BDQ and 21 received DLM as a background regimen.

Minimum sample size determination procedure for estimating a population proportion was adopted here. The formula used for the purpose is as follows: $(z_{1-\alpha /2}^{2} P(1-P)/d^{2})$. Pilot analysis of previous data found that the non-adherence was about 30% and adherence was about 70%. In this study following values of the above parameters have been considered keeping in view the frequency of availability of the cases in the study hospital (confidence level=1-∝ =95%, anticipated population proportion p=70%, and d=10%). The minimum sample size required is computed at 81. However, we have a sample of 86. Data collected on 86 cases were entered into SPSS version 24.0 (Karnataka, India: SPSS South Asia Private Ltd.). The statistical analysis was done using frequency distribution procedure, chi-square test of independence. Comparison of mean number of ADR between drug groups was made by using an independent sample t-test. The significance level was set at p<0.05.

## Results

Table 1 shows the demographic distribution of the study population. All patients in the study were from both rural and urban areas. Patients were mostly of the middle age group with mean age of 35.2 years. Male patients outnumbered female patients. The maximum number of patients while receiving treatment were married and not working. The education level was greater than 10th grade in the majority of the population. Patients' socioeconomic status (SES) was classified as per the BG Prasad scale. The majority of the patients belonged to lower SES status. The total number of patients co-infected with HIV was very less. Majority of the patients were previously treated for TB.

**Table 1:** Demographic distribution of the study population. SES: socioeconomic status

Variable		Frequency	Percentage (%)
Gender	Male	67	78
	Female	19	22
Marital status	Married	47	54.9
	Unmarried	39	45.1
Educational status	Literate	65	75.8
	Illiterate	21	24.2
Currently working	Yes	17	19.9
	No	69	80.1
Socioeconomic class (SES)	Low SES	51	59.3
	Medium SES	25	29.1
	High SES	10	11.6

The analysis of spectrum of ADRs is presented in Table 2. ADR is the most challenging aspect in the treatment of TB, and this may also lead to non-adherence. It was revealed that there were as many as 24 types of ADRs manifested in different patients. The most frequent ADR was QTcF (corrected heart rate) prolongation which occurred among 29 cases (33.7%). This was followed by vomiting (23 cases, 26.7%), vertigo (11 cases, 12.8%), arthralgia (10 cases, 11.6 %), and weakness (10 cases, 11.6%). Other types of ADRs were psychosis, electrolyte imbalance, hyperpigmentation of face, skin rash, headache, peripheral neuropathy, and nephrotoxicity which occurred in five to nine cases. The remaining ADRs were hearing loss, giddiness, metallic taste, hepatitis, depression, itching, suicidal ideation, blurred vision, hypothyroidism, and tremor which occurred in less than four cases. Death was seen in 12 cases (14.0%). None of the ADRs had significant association with the drug type (p>0.05).

**Table 2:** Spectrum of ADRs of both groups. ADR: adverse drug reaction; QTcF: corrected heart rate; MDR multidrug-resistant; XDR: extensively drug-resistant

No. of ADR	MDR/XDR				Total		Chi-square p-value
	Bedaquiline		Delamanid				
	(n=65)		(n=21)				
	No.	%	No.	%	No.	%	
QTcF prolongation	21	32.3	8	38.1	29	33.7	0.626
Vomiting	15	23.1	8	38.1	23	26.7	0.176
Vertigo	9	13.8	2	9.5	11	12.8	0.606
Arthralgia	7	10.8	3	14.3	10	11.6	0.662
Weakness	6	9.2	4	19	10	11.6	0.222
Psychosis	5	7.7	4	19	9	10.5	0.139
Skin rash	4	6.2	4	19	8	9.3	0.077
Hyperpigmentation face	6	9.2	2	9.5	8	9.3	0.968
Gastritis	6	9.2	2	9.5	8	9.3	0.968
Electrolyte imbalance	6	9.2	2	9.5	8	9.3	0.968
Headache	3	4.6	3	14.3	6	7	0.13
Peripheral neuropathy	5	7.7	1	4.8	6	7	0.647
Nephrotoxicity	3	4.6	2	9.5	5	5.8	0.403
Hearing loss	3	4.6	1	4.8	4	4.7	0.978
Metallic taste	2	3.1	1	4.8	3	3.5	0.714
Giddiness	2	3.1	1	4.8	3	3.5	0.714
Depression	2	3.1	0	0	2	2.3	0.416
Suicidal ideation	2	3.1	0	0	2	2.3	0.416
Hepatitis	1	1.6	1	4.8	2	2.4	0.401
Itching	2	3.1	0	0	2	2.3	0.416
Hypothyroid	1	1.5	0	0	1	1.2	0.568
Tremor	0	0	1	4.8	1	1.2	0.077
Blurred vision	1	1.5	0	0	1	1.2	0.568
Death	8	12.3	4	19	12	14	0.438

Table 3 present the comparison of mean number of ADRs between the drug groups. In the BDQ group, the average number of ADR per patient was 1.7±1.5 while in that of the DLM group was 2.4±1.4. But the difference was not statistically significant (p=0.080). Overall there were 17.4% of cases where there were no ADR. The said proportion was 21.5% in BDQ group and 4.8% in DLM group. Among 32.6% of cases there was one ADR while in 50% of cases there were two or more ADRs. The ADR didn’t have significant association with the drug group.

**Table 3:** Comparison of number of ADRs between drug groups. *Independent sample p-value. BDQ: bedaquiline; DLM: delamanid; SD: standard deviation; ADR: adverse drug reactions; MDR: multidrug-resistant; XDR: extensively drug-resistant

No. of ADR	MDR/XDR				Total		p-Value
	BDQ		DLM				
	No.	%	No.	%	No.	%	
0	14	21.5	1	4.8	15	17.4	χ2=4.669, p=0.458
1	22	33.8	6	28.6	28	32.6	
2	12	18.5	5	23.8	17	19.8	
3	6	9.2	4	19	10	11.6	
4	7	10.8	3	14.3	10	11.6	
5	4	6.2	2	9.5	6	7	
Total	65	100	21	100	86	100	-
Mean±SD	1.72±1.49		2.38±1.43		1.88±1.50		0.080*

Causality assessment of ADRs obtained with World Health Organization-Uppsala Monitoring Centre (WHO-UMC) criteria was categorized into certain, probable, and possible. For patients who were receiving BDQ as optimized background regimen, the ADRs were maximally categorized in the possible group (68%). The number of patients who were categorized into certain and probable groups was 18.6% and 13.4%, respectively. Similarly, in the DLM group, majority of the patients, (57.3%) were categorized into the possible group. The number of patients who were categorized into certain and probable groups was 10.3% and 32.4%, respectively (Table 4). The certain ADRs were QTc prolongation, hearing loss, blurring of vision, and suicidal ideation in both groups.

**Table 4:** WHO-UMC causality scale for assessment of ADRs. BDQ: bedaquiline; DLM: delamanid; WHO-UMC: World Health Organization-Uppsala Monitoring Centre; ADR: adverse drug reaction

Assessment scale	BDQ		DLM		Total		χ2, p-value
	No.	%	No.	%	No.	%	
Certain	12	18.6	2	10.3	14	16.6	χ2=4.290, p=0.117
Probable	9	13.4	7	32.4	16	18.0	
Possible	44	68	12	57.3	56	65.4	
Total	65	100	21	100	86	100	-

Eighty-six patients' ADRs were analyzed for severity assessment using the Siegel scale which was categorized into mild, moderate, and severe. In the BDQ group, majority of the ADRs were mild (61.7%) whereas 25.5% and 12.8% were moderate and severe, respectively. In the patients receiving DLM as background regimen, 69.4% of ADRs were categorized as mild, whereas 14.5% and 16.1% were categorized as moderate and severe, respectively (Table 5).

**Table 5:** Modified Hartwig Siegel severity scale for assessment of ADRs. BDQ: bedaquiline; DLM: delamanid; ADR: adverse drug reactions

Assessment scale	BDQ		DLM		Total		χ2, p-value
	No.	%	No.	%	No.	%	
Mild	40	61.5	15	71.4	55	63.9	χ2=1.253, p=0.535
Moderate	17	26.1	3	14.3	20	23.2	
Severe	8	12.3	3	14.3	11	12.8	
Total	65	100	21	100	86	100	-

## Discussion

To ensure a successful cure, individuals with TB should adhere to their treatment regimen at a rate of at least 90%. When system isn't working, there's a greater chance that drug-resistant strains will develop and that TB will spread across the population, which raises mortality and morbidity rates [9]. The present study is a vivid analysis of ADR in pre-XDR and XDR TB patients. The findings of this study need to be validated against the findings of similar studies conducted at different times and spaces to draw insight and its implications for treatment management. The present study has a sample size of 86 and mean age of 35.71±12.63 years. The sample is quite young and nearly three-fourth are males. In a similar study conducted in Sub-Saharan Africa by Young et al. showed that majority of the samples were female which is not in accordance with our study, but the mean age group finding was similar with their study. A total of 78.1% of cases were having low level of education [10,11]. One-fourth of the patients were illiterate and another quarter were having education up to primary level. These implied illiterate patients were more affected by TB. These associations have previously been documented in other Sub-Saharan African countries, but this is the first research of its kind in Odisha [3,12]. In contrast to the results of a study conducted in Equatorial Guinea, the majority of the patients were not working throughout the disease period [13]. A total of 89.7% of sample population had previous history of treatment for MDR TB. This signifies that these patients previously were defaulters or had treatment failure or discontinued treatment for other reasons for which they have developed now pre-XDR and XDR TB. Researchers Gube et al. did a similar study at Arba Minch governmental health institutions in Southern Ethiopia and found similar results [14]. Drugs used in pre-XDR and XDR TB therapy have been shown to have a well-documented history of adverse events. Circumventing ADR is the most challenging aspect in the treatment of TB, 24 types of ADRs were manifested in different patients in this study. The most frequent ADR was QTcF prolongation (33.7%) followed by vomiting (26.7%), death (14.0%), head reeling (2.8%), arthralgia (11.6%), and weakness (11.6%). Other types of ADRs were psychosis, electrolyte imbalance, hyperpigmentation of face, skin rash, headache, peripheral neuropathy, nephrotoxicity, hearing loss, giddiness, metallic taste, hepatitis, depression, itching, suicidal ideation, blurred vision, hypothyroidism, and tremor. In the BDQ group, the average number of ADR per patient was 1.7±1.5 while in that of the DLM group was 2.4±1.4. But the difference was not statistically significant p=0.866. Overall there were 17.4% of cases where there was no ADR. In our study, QTcF prolongation was the most common adverse reaction. All patients were receiving various QTcF-prolonging medications, including BDQ, DLM, clofazimine, moxifloxacin, or levofloxacin, in combination. BDQ has been linked to similar patterns of adverse drug reactions in previous trials. Compared to placebo, BDQ had a higher chance of prolonging the QTcF in the phase 2 randomized controlled trial of five additional anti-tuberculosis medications [15]. BDQ and DLM were also linked to an elevated risk of mortality that was not explained in this investigation. Two out of 233 individuals on BDQ exhibited clinically significant QTcF prolongation (>500 ms) in an open-label, single-arm observational research. Both patients were also taking clofazimine concomitantly. In light of these research, the FDA issued a black box warning about the elevated risk of sudden death and QTcF prolongation in the prescription instructions [16].

Similar to earlier studies, we found that the second most prevalent adverse event was gastrointestinal symptoms (nausea, vomiting, and moderate gastritis) [16,17]. Gastrointestinal (GI) symptoms may be caused in part by quinolones and ethionamide, according to research. The symptoms were mild, yet they need rapid intervention. The majority of these gastrointestinal side effects appeared within a week after beginning treatment, however, some persisted for longer periods. A causality investigation identified quinolones and ethionamide as the most likely culprits. Administering these medications one hour after taking a domperidone and proton pump inhibitor pill can help prevent these adverse drug events. The next most prevalent ADRs were arthralgia and psychosis. Pyrazinamide and quinolones appeared to be related to arthralgia. The arthralgia and arthritis that pyrazinamide causes are due to an increase in blood uric acid levels, whereas quinolones destroy cartilage [18]. As in prior investigations, cyclosporine was found to have a clear connection to psychosis and suicidal ideation [19]. It happened within the first six months of therapy. PAS was initially used to treat these people. An increase of 1.5 times in pre-treatment alanine transaminase (ALT) values indicates hepatotoxicity. Pyrazinamide and BDQ-induced hepatotoxicity appear to have a possible causal link. Anti-TB medications that cause hepatotoxicity most often do so by causing toxic metabolites to develop or be removed more slowly, resulting in abnormalities in test values within two to three months. According to recent research, genetic factors, or polymorphism of drug-metabolizing enzymes, play an essential role in hepatotoxicity, which is facilitated by drug dosage [20]. Within seven to two months of commencing treatment, a localized erythematous rash appeared. Quinolones and pyrazinamide appeared to be related in some way. Pyrazinamide-induced rashes occurred in 0.1-5% of cases [21-23]. While we discovered modest erythematous reactions in our study, they did not need a change in the drug regimen. Seven months after starting the drug treatment, peripheral neuropathy of the sensory kind developed [24].

There is a high correlation between ethionamide and this disease. Kanamycin may have played an important role. Tubular cytotoxicity is the initial effect of aminoglycosides; after that, lower glomerular filtration is generated by vascular and mesangial contraction, leading to aminoglycoside tubular cytotoxicity. All aminoglycosides, including kanamycin, are known to cause vestibulotoxicity [25]. NMDA receptor stimulation or iron binding are the two mechanisms by which aminoglycosides cause an increase in free radicals [26]. It was definitely connected to ethambutol in some way [27]. Excessive levels of extracellular glutamate may make ganglion cells more sensitive to excitotoxic toxicity, according to some research [28]. One male patient had hypothyroidism by the end of the fourth month after starting medication. Ethionamide was the culprit in a hypothyroidism case, and there was a high probability that the two were linked. Propylthiouracil and methimazole are both thioamides, therefore ethionamide might limit thyroid hormone production by an analog mechanism of iodine organification inhibition [29]. One of the unusual ADRs was tremor, which wasn't on the list of ADR drugs. A total of 14% of the patients in the trial group died. Multiple factors contributed to the death of the patients. The cause of the disease was not identified. To keep an eye out for treatment-limiting side effects, pharmacovigilance procedures should be promoted and supported within drug-resistant rehabilitation programs.

## Conclusions

BDQ and DLM-containing therapy as an optimized background regimen increases cure rate and reduces mortality. This study showed that the most common ADR was QTcF prolongation followed by other adverse effects, such as vomiting, arthralgia, hearing loss, weakness, and suicidal ideation. The majority of ADRs were mild and had a possible relationship with the suspected drugs. Robust data on the incidence and prevalence of serious ADRs in patients receiving BDQ and DLM containing TB treatment, and QTcF prolongation, in particular, is a research priority to guide programmatic management for optimal monitoring of patients additionally all the patients should be patiently explained regarding the potential adverse effects of BDQ and DLM before initiation of therapy.
